# The role of physical activity in cancer survivors’ quality of life

**DOI:** 10.1186/s12955-020-01448-3

**Published:** 2020-06-22

**Authors:** Tayah M. Liska, Angela M. Kolen

**Affiliations:** 1 Department of Human Kinetics, St. Francis Xavier University, 1140 Convocation Blvd, Antigonish, NS B2G 2W5 Canada; 2 Present Address: Department of Kinesiology and Physical Education, McGill University, Montreal, Québec Canada

**Keywords:** Exercise, Interviews, Qualitative research, Mental health

## Abstract

**Purpose:**

As a result of a cancer diagnosis and treatment, many cancer survivors experience persistent physical, mental, and emotional symptoms that affect their quality of life. Physical activity has been identified as an intervention that may help to manage the side effects of a cancer diagnosis and its treatment. The purpose of this study was to investigate the role of physical activity on overall quality of life in adult cancer survivors.

**Methods:**

One-on-one semi-structured interviews were conducted in person or via telephone with 13 adult (≥18 yrs) cancer survivors who had completed cancer treatment.

**Results:**

These cancer survivors described their physical activity as improving their physical functioning and mental health, as a means of positive social engagement, and adding positivity to their daily life.

**Conclusion:**

Even though the cancer survivors in this study experienced diverse types of cancer and for different lengths of time as well as varying methods of treatment(s), these results support the role of physical activity in enhancing cancer survivors’ quality of life. Further research is warranted to (a) expand this research with a larger sample and quantitative methods, (b) examine healthcare providers’ knowledge and application of exercise guidelines to cancer survivors in cancer care, and (c) explore implementation strategies for greater advocacy for healthcare providers to share the exercise recommendations with cancer survivors.

## Background

Each year, over 200,000 Canadians are diagnosed with cancer [1]. Due to improvements in early detection and treatment, 60% are expected to survive their disease for five or more years, and this percentage is expected to rise [1]. Following cancer treatment, health care providers monitor their patients for cancer recurrence and the late and long-term effects of cancer, which is now considered a chronic illness. Recently that the long term health of cancer survivors has been given consideration [2, 3]. The National Cancer Institute considers an individual a cancer survivor from the time of diagnosis, through the balance of his or her life [4]. For the purpose of this research project, specific attention was given to cancer survivors who by definition are those who completed their active cancer treatment (i.e) surgery, chemotherapy, and/or radiation.

Many cancer survivors experience persistent, physical, mental, and emotional changes that affect their quality of life (QoL) [5]. Quality of life refers to one’s sense of wellbeing and encompasses a multidimensional perspective that includes physical, emotional, and spiritual domains [6]. Physical activity (PA)^1^ is an intervention that might help manage the side effects of a cancer diagnosis and treatment, as well as improve the QoL for cancer survivors [7]. Engaging in regular PA improves muscular strength and cardiovascular endurance, increases overall energy levels and the physical capability to complete daily tasks, and reduces risk for chronic diseases [5, 8, 9]. Segal et al. [7] reported positive influences for physical and mental health in cancer survivors who engaged in an exercise intervention during treatment or post-treatment. Physiologically, PA can be effective in mitigating the cluster of symptoms caused by cancer treatment at their biologic origins [9]. Research indicates that regular PA or exercise stimulates positive psychological functioning and can be used as a method for coping with side effects of cancer and its treatment such as increased feelings of depression, anxiety, sleep difficulties and cancer related fatigue, and the cognitive confusion or impairment that often persists after cancer treatment [5, 10–14].

The physical and mental health and well-being benefits of exercise may be even greater when involved in group exercise interventions as noted by Martin et al. [15] for individuals with breast and prostate cancer. In individual and focus group interviews, breast and prostate cancer survivors reported that the group environment of their exercise classes allowed them to feel a sense of belonging, normality, mutual aid, and bonding, as well as an opportunity for sharing coping and success strategies [15]. These findings suggests that support groups that include PA may benefit the cancer survivors’ personal well-being, QoL and serve as a coping tool to manage feelings of personal stress and emotion [5, 7, 15].

Livsey and Lewis [16] noted that cancer survivors who engaged in meaningful group PA experienced greater feelings of confidence, comradery, friendship, and empowerment. These feelings were more profound and persisted longer when the group participants were primarily fellow cancer survivors [5, 15, 16]. Furthermore, cancer survivors who had greater motivation, sense of inclusion, and empathic understanding of fellow survivors reported higher adherence to PA [5]. The increased positive emotions, physical and mental relief from stress, and improved mental health from group exercise further highlight the importance of PA and its role in improving cancer survivors’ QoL.

Although our understanding of the mental and physical health benefits of regular PA for cancer survivors is increasing, less is known about cancer survivors’ perspectives of their PA through their lived experiences of cancer and its treatment. Therefore, the purpose of this study was to learn more about PA in adult cancer survivors and how it influenced their physical and mental health and overall QoL.

## Methods

A phenomenological approach was used to guide the data collection and analysis in this study. This approach allowed the researchers to learn from the participants and their lived experiences regarding their physical activity, cancer, and cancer treatment [17]. This approach was chosen as it is believed that only those who live the experience can describe it [17]. Following Research Ethics Board approval and informed consent, one-on-one in-depth semi-structured interviews with adult (≥18 yrs) cancer survivors who completed cancer treatment were conducted. Participants were recruited from Nova Scotia, Canada via advertisements in local and provincial newspapers. Individuals interested in participating in this study contacted the first author via email or telephone. Following an introductory conversation, sharing of the letter of invitation, and obtaining written informed consent, interviews were conducted by the lead author in January and February 2018 based upon the participants’ availability. These interviews were conducted in-person (*n* = 4) when the participant lived nearby and over the phone when they did not (*n* = 9). Regardless of how the interviews were conducted, the interviewer engaged in small talk to build rapport to facilitate the sharing of personal experiences. Interviews were audio-taped and averaged 40 min in duration with a range of 15 to 60 min.

Both authors are trained in qualitative research methods, and the lead author conducted one pilot interview with a cancer survivor prior to data collection. Semi-structured interviews are often used in health-related and physical activity research as they provide opportunities for elaboration about specific life experiences [18]. Table 1 identifies the guiding and probing questions participants were asked about their lived experience with their cancer diagnosis and cancer journey, including their treatment(s), side effects, and their PA prior to and following their cancer diagnosis and treatment.

**Table 1:** Interview Guide

1. What was your cancer diagnosis? The disease site and stage of cancer, and how long has it been since your diagnosis?	
2. Please explain/describe/share the cancer treatment(s) that you received?	
3. How long has it been since you completed your cancer treatment and been considered a survivor?	
4. Could you describe your daily life prior to your cancer diagnosis? - Dietary intake? Stress? PA?	
5. How would you describe your social network, such as family support and relationship with friends? - Was it similar during and after treatment? How were these relationships impacted by your cancer treatment?	
6. Were you physically active *prior* to your cancer diagnosis? - How often? How long? What activity did you do? Planned vs. unstructured?	
7. In what way did your cancer treatment impact you? Physically, mentally, emotionally?	
8. Were you physically active during your cancer treatments? - If active, do you think it may have helped you physically, mentally, or emotionally cope with cancer treatment?	
9. What was your daily life like after being diagnosed with cancer? Please explain. How was your daily life impacted by your cancer diagnosis? - Routines, seeing family and friends, being physically active.	
10. Have any of the physical, mental, or emotional feelings that you experienced while undergoing cancer treatment continue after your treatment was complete?	
11. Are you aware of the Canadian Physical Activity Guidelines, or has anyone brought them your attention?	
12. Did your health care provider recommend for you to be physically active *during* the course of your cancer treatment? A*fter* the completion of your treatment? - What was their recommendation? - Do you feel the topic of physical activity is something of interest or importance for your health care provider to speak to you about? Should health care providers talk about physical activity to individuals during cancer treatment?	
13. Do you think physical activity could, in general, provide benefits to your overall daily life? If so, could you explain how? In what way might physical activity benefit your daily life? - the physical and mental feelings that you may experience after being physically active. (Energy levels, mood, strength). - the possible change in physical and mental feelings after being physically active (energy levels, mood, strength).	

### Data analysis

Using phenomenology to guide the data analysis, each audio recording was listened to, reviewed, and analyzed through a journaling and transcription process. Interviews were transcribed by the lead author. To assist with methodological rigor, completed transcriptions were shared with the participants for their approval/confirmation and to provide an opportunity to elaborate or modify comments made [19, 20]. Once the transcripts were complete, the authors reviewed and coded them. When disagreement of the coding arose, the authors engaged in a collaborative discussion to resolve the discrepancy and reach an agreement that best reflected the data. Once the interviews were coded, the authors continued analyzing the data allowing the most prevalent themes to emerge through discussion and deliberation.

## Results

Twenty people expressed interest in participating in the study with thirteen (5 male; 8 female) individuals providing data. Details related to their cancer, treatment, side effects, and physical activity are shared in Table 2. It should be noted that what is reported in this table is based on the participants’ lived experiences and does not represent their medical diagnosis or treatment. Similarly, the physical activity levels included in this table are based upon the participants’ self-report and interpretation versus an objective measurement.

**Table 2:** Cancer treatment and physical activity of 13 cancer survivors

Participant^a^	Cancer Diagnosis	Treatment(s)	Duration of Cancer Treatment	Side Effects from Treatment	PA Level^b^ Prior to Diagnosis	PA Level After Treatment	Types of PA
Marie	Invasive ductal carcinoma (breast cancer) ER positive, HER2/NEU negative	Mastectomy Prophylactic hysterectomy 8 cycles chemotherapy 25 radiation treatments	**Diagnosis**: July, 2005 **Completion**: March, 2007	Nausea Joint pain Fatigue Low immune system Inability to sleep Hair loss High FSH	Very high	Very high	Walking Yoga Weight training Golf Aerobic classes Pickle ball
David	Chronic lymphocytic leukemia	Chemotherapy	**Diagnosis** 2006 **Completion** 2009	Low hemoglobin level Vomiting immediately after chemo	Very high	Very high	Walking Running Tennis Hockey
Mark	Colon cancer	Surgery, resection of the lower bowel 8 cycles chemotherapy 25 radiation treatments	**Diagnosis** May, 2017 **Completion** August, 2018	Recovery from surgery Lack of energy Hiccups Chemo-induced-peripheral- neuropathy High sensitivity to cold	High	High	Walking Outdoor activity
Catherine	Breast cancer	Surgery 4 cycles chemotherapy 25 radiation treatments	**Diagnosis** November, 2016 **Completion** June, 2017	High blood pressure Fatigue Hair loss Chemo burn	Moderate	Moderate	Walking Swimming Aqua fit classes
Kelly	Small bowel cancer	Surgery 12 cycles chemotherapy	**Diagnosis** February, 2018 **Completion** January, 2019	Hand and foot syndrome Blocked tear ducts Fatigue	High	Moderate	Walking Aerobics classes Gym
Graham	Non-Hodgkin’s Lymphoma	5–6 months of chemotherapy One month radiation	**Diagnosis** May, 1995 **Completion** March, 1996	Hair loss Nausea	Moderate	Moderate	Gym Swimming Running
Patrick	Prostate cancer	Surgery	**Diagnosis** March, 2018 **Completion** May, 2019	Recovery from surgery Weaken of pelvic floor from surgery	High	High	Hockey Gym Walking
Grace	Invasive ductal carcinoma (i.e., breast cancer) Estrogen Positive HER2 Positive	4 cycles chemotherapy 16 radiation treatments	**Diagnosis** December, 2015 **Completion** March, 2017	Hair loss	Moderate	Moderate	Walking Swimming
Claire	Breast cancer	Surgery Radiation Chemotherapy	Treatment received 10 years ago.	Radiation burns Fatigue Nausea Illness	Moderate	Moderate	Walking Swimming Aqua fit classes
Jane	Breast cancer	Mastectomy 4 cycles chemotherapy	**Diagnosis** October, 2016 **Completion** Fall, 2018	Change in heart beat proficiency Allergic reaction to chemo-therapy Headaches Body Aches Hair loss	Moderate	Moderate	Walking Skiing Biking Volleyball Squash Aqua Fit Gym
Anna	Breast cancer	Double mastectomy 6 cycles chemotherapy 25 radiation treatments	**Diagnosis** March, 2016 **Completion** Early 2017	Radiation burns Low white blood cell count Memory difficulty Skin sensitivity	Low	Moderate	Walking Aqua fit classes
Lisa	Primary mediastinal large B-cell lymphoma	6 cycles chemotherapy 25 radiation treatments	**Diagnosis** June, 2018 **Completion** January, 2019	Hair loss Body pain Bone pain Fatigue Cognitive confusion Neuropathy Exhaustion	Moderate	Low	Walking Outdoor activities and play with kids
Andrew	Prostate cancer	Surgery	**Diagnosis** May, 2017 **Completion** May, 2017	Recovery from surgery Weight loss Vomiting	Moderate	Low	Walking Running Swimming Basketball

^a^Pseudonyms

^b^PA Level = Physical activity self-reported, where very high = at least an hour each day; high = between 30 and 60 min each day, moderate = about 30 min each day; low = less than 30 min each day

The ongoing reflection of the data during collection and analysis provided insight into the commonalities among the participants as they reflected on their cancer journeys, their physical activity and its influence on their QoL. Key quotes and statements were highlighted, organized and categorized based on the three main themes that arose from the analysis; physical benefits, psychosocial benefits, and continuing cancer care. These themes, the quotes and lived experiences within them, were then individually and collectively connected to participants’ QoL.

### Physical benefits

Four participants described having little or no long-term side effects from their cancer treatment. These participants believed their regular PA prior to cancer treatment and, in some cases, during their cancer treatment was the reason for the non-existence or limited side-effects. To exemplify, when Grace was asked why she thought she did not experience side effects, she answered “I really do attribute the fact that I went through treatment so easily to the fact that I was so active”. Similarly, Kelly reported she believed PA helped her manage the stomach and pelvic pain she experienced during and shortly after completing treatment.

Participants also reported of the benefits they believed they experienced from their PA since completing treatment. Most intriguing in this regard was Marie, a breast cancer survivor. She had been taking an oral endocrine therapy to reduce the risk of cancer reoccurrence that has the potential side effect of reducing bone density. When Marie learned resistance training improves bone density, she added it to her exercise regime. Marie shared,

“My bone density actually improved. That’s when I began a really rigorous exercise program. Sort of at that time I was doing research on how to improve my bone density and exercise was one of the things. So, I actually began doing weights”.

Another example is Catherine. Although she took a prescription drug that exacerbated her rheumatoid arthritis, she continued to engage in PA because she believed she needed it to maintain/improve her current physical health and recovery from cancer treatment.

Concurrent with the belief that PA provided physical benefits after cancer treatment, participants also spoke specifically about how PA facilitated their recovery process. Participants alluded that they felt stronger and in some instances their recovery was easier because they were routinely physically active. Most notably, the participants indicated the value of PA for pain management post-treatment, for reducing further physical loss following surgery, and as part of their rehabilitation. Overall, the participants were emphatic in expressing the importance of regular physical activity for them to help manage the late or long-term effects from their cancer treatment. This notion is directly supported by Patrick’s statement; “my belief is that physical activity helps the body heal. I think it’s also good for the mind, which in turn helps the body heal … I think physical activity is an important part of the healing process”.

### Psychosocial benefits

Participants described mental health benefits or feelings of self-gratification from their PA. This positive mental well-being was considered equally important to the survivors as the physical health benefits they experienced from their PA. Participants noted that their PA was something to look forward to as it allowed them to get up and out of the house and to interact with others. Claire spoke highly of her PA as a positive coping mechanism for the mental strain she felt while recovering from her treatment, reducing these feelings of mental strain as well as those from other personal stresses at the time. When Grace reflected on her lifestyle change when first diagnosed with cancer and in particular becoming physically active on daily basis, she spoke highly of the increase in her energy and mood while undergoing treatment. In a confident and appreciative tone, Grace shared that PA had become important and personally valuable to her mental wellbeing both during and after cancer treatment. Grace illustrated the effect of PA for her in the following; “Oh definitely. I don’t think I realized it initially that that was doing it. Even the sky was bluer if you could imagine that … It was probably the worst year of my life, but it was also the best year of my life.” When asked if these feelings persisted after completing treatment, she strongly agreed that PA had made a lasting, significant difference on her mental and physical health. Similarly, others said it was not just because it was important for their physical health but rather, they engaged in PA because it was enjoyable and benefitted them personally. David stated in a happy tone; “it’s gone beyond the point of it being a chore, it’s what we normally do”. This statement was in addition to David’s insightful perspective on how PA provided him with a sense of elation and satisfaction towards his personal wellbeing.

### Continuing cancer care

Regarding conversation with their healthcare team regarding PA, most participants said these were casual where their physician(s) may have asked if they were physically active and what types of PA they may be doing. Participants were then asked if they thought their physician’s awareness of their previous or current engagement in PA influenced these conversations. Most participants felt that since their physician(s) were aware of their physically active lifestyle, conversations about PA were not required. Still, these participants believed personal conversations related to PA, or physicians’ affirmation of their PA, were beneficial and necessary.

Two participants reported difficulty initiating conversations about PA with their physician. It was only when they specifically asked about an acceptable level of intensity for their PA that a short conversation occurred. Another participant reported feeling unsure about how to introduce PA into her daily life shortly after completing cancer treatment. Conversely, four participants shared their experience with their oncologist who was adamant that they should be participating in PA. These participants spoke of how these appointments with their oncologist included a descriptive conversation about what PA they were doing and they were explicitly encouraged to be as physically active as possible. Finally, participants were explicitly asked about the *Cancer Care Exercise Guidelines* [21] in their interviews to determine if they were provided to cancer survivors following completion of their cancer treatment. No participant was aware of the *Cancer Care Exercise Guidelines* [21]. However, it should be pointed out that during the discussions about receiving, or being aware of, cancer care exercise guidelines, participants mentioned receiving copious reading material upon completion of their treatments. Participants suggested some of this literature may have included information about PA, but they could not recall whether it was actually included.

## Discussion

The purpose of this study was to learn more about the lived experiences of cancer survivors related to their physical activity pre- and post-cancer treatment and its effect on physical and mental health and their overall QoL. Despite the different post-cancer experiences amongst the participants, the commonalities and connections of improved physical and psychosocial wellbeing from their engagement in PA became apparent. In fact, all participants talked of their belief in the connection between their PA and improved QoL, regardless of the intensity of treatment(s) experienced and the time since completion of their treatment. As shared previously, these participants provided elaborate details regarding their perceptions of their improved physical wellbeing, recovery time, and ability to complete daily tasks from their PA. Furthermore, survivors communicated that the introduction or re-establishment of PA into their daily routine led to a positive recovery and increase in physicality after the completion of their cancer treatment. Most commonly, participants described their PA as a means of positive management of their health with an expressed desire to remain physically healthy and to reduce their risk of cancer reoccurrence most evident. As such these participants noted a continuous effort to either maintain or adopt regular PA into their daily lives.

Participants who experienced ongoing stress stated their awareness and understanding of the benefits and enjoyment of PA. With this, participants provided a variety of supporting explanations of improved mood and energy levels, and positivity after engaging in PA. In particular, being active outside in the fresh air and participating in familiar PA provided mental health benefits and a reduction in personal stress. These positive psychological factors provided reinforcement for regular PA and helped with adjustments experienced post-treatment. It should be noted that these reports of mental and emotional improvement in the participants’ lives were described with elation and positive emotions overall.

During their interviews, participants were asked if PA was a routinely discussed with their physician(s).

The variation in reported conversations with physicians does not suggest a particular direction physicians’ take in having conversations regarding PA with their patients. Also, the variance in participants’ responses does not indicate if physicians provide PA prescriptions or discuss the PA guidelines with their patients. This raises questions regarding the importance of promoting the exercise guidelines in cancer care. Further, it raises an important question as to whether exercise guidelines should be integrated into the standard of care of health care provider clinical practice by routinely providing PA prescriptions. Nonetheless, the connection between PA promotion and PA engagement by cancer survivors should not be overlooked. The positive physical and psychosocial responses provided by participants further suggest that PA plays a role in survivors’ QoL. Understanding this, coupled with an awareness of that published clinical guidelines exist on exercise for people with cancer [21], it should be suggested that physicians express the benefits of regular PA to their patients. Further, the verbal conservations that participants reported having with their physicians in this study serves to support this as an important ongoing strategy for cancer survivors to initiate or sustain regular PA, and offer potential for improving cancer survivors QoL.

Each cancer survivor reported improved QoL from their engagement in PA, despite individual differences in cancer treatment(s), duration, or time from the end of their active cancer treatment. This improved QoL was unique to each person and was noted more than once in their expressions of the physical and psychosocial benefits they experienced from their participation in PA. Considering the study’s operational definition of QoL recognizes the multidimensional perspectives that includes physical, emotion, and spiritual domains [5], the lived experiences expressed by participants indicates that PA participation influences QoL, to some capacity, at an individual level.

Although heterogeneity and sample size of the participants within this study allowed for diversity and richness in the collection of robust, qualitative data, it is recognized that the responses and personal experiences were individualistic. It is also important to point out that the participants shared their lived experiences with their physical activity, cancer diagnosis, treatment and side effects. As such the data reported in Table 2 were not objective, but rather subjective and presented in the way that the participants recalled their experiences. Further research is warranted with a larger sample size with further exploration of patient knowledge and experience using quantitative methodologies. Further, it may be important to examine healthcare providers’ knowledge of the exercise guidelines and their recommendations for use with cancer survivors.

## Conclusion

Each cancer survivor reported specific lived benefits experienced from their PA participation. Upon examination of the different cancer diagnoses, treatment, and post-cancer experiences amongst participants, similar clustered themes of improved physical and mental health and well-being from PA were shared. Thus, the results from this phenomenological, qualitative analysis indicated that adopting a lifestyle that includes routine PA after cancer treatment is beneficial for cancer survivors and their overall QoL.

## Data Availability

The one-on-one interview data collected and analysed in the current study are available from the corresponding author on reasonable request.

## Abbreviations

PA: physical activity
QoL: quality of life

## Footnotes

1 In the context of this study, PA is defined as moderate to vigorous intensity activities that increases a person’s energy expenditure and heart rate. Exercise refers to a structured, intentional form of PA. Given that cancer survivors used PA and exercise in the same context, these terms are also used interchangeably throughout this research paper.
